# Personal strengths reported by people with chronic illness: A qualitative study

**DOI:** 10.1111/hex.12674

**Published:** 2018-02-25

**Authors:** Olöf Birna Kristjansdottir, Una Stenberg, Jelena Mirkovic, Tonje Krogseth, Tone Marte Ljoså, Kurt C. Stange, Cornelia M. Ruland

**Affiliations:** ^1^ Center for Shared Decision Making and Collaborative Care Research Division of Medicine Oslo University Hospital Oslo Norway; ^2^ Norwegian National Advisory Unit on Learning and Mastery in Health Oslo University Hospital Oslo Norway; ^3^ Department of Pain Management and Research Oslo University Hospital Oslo Norway; ^4^ Center for Community Health Integration Case Western Reserve University Cleveland OH USA; ^5^ Faculty of Medicine Institute of Clinical Medicine University of Oslo Oslo Norway

**Keywords:** chronic illness, personal strengths, health assets, self‐management, salutogenesis, empowerment

## Abstract

**Background:**

Self‐management of chronic illness can be highly demanding and people need to mobilize their personal strengths to live well with their condition. More knowledge is needed about how people with chronic illness perceive and use their personal strengths as a basis for better integrating empowering person‐centred approaches into health care.

**Objective:**

To explore what people with chronic illness describe as their strengths relevant to their health and well‐being.

**Setting and Participants:**

Thirty‐nine participants (11 men) from 4 outpatient self‐management programmes were recruited to individual or group interviews. Participants included patients with chronic respiratory disease (n = 7), chronic pain (n = 18) and morbid obesity (n = 14). Interviews were analysed using content analysis.

**Results:**

A number of personal strengths were reported and categorized into 3 domains: (i) Internal strengths, (ii) External strengths and (iii) Self‐management strategies. Internal strengths included being persistent, having a positive outlook, being kind and caring, experiencing positive emotions, being kind towards oneself, reconciling oneself with the situation, having courage and having knowledge and insight. External strengths included support from family, friends, peers and health‐care providers. Self‐management strategies included being active, planning and prioritizing, reducing stress, goal setting and seeking knowledge and help.

**Discussion and Conclusion:**

The study provides insights into personal strengths as reported by people with chronic illness. The results complement prior findings on strengths in people with health challenges and can aid in incorporating person‐centred approaches into health care.

## 1 INTRODUCTION

Self‐management involves the tasks people with chronic illness must undertake to live well with their condition.^1^ These tasks can be demanding, as people need to learn about their condition and medications, manage their symptoms and emotions, as well as making recommended lifestyle changes.^1^ An essential process to learning to live well with chronic illness is to find and use one's own strengths or health assets.^1^, ^2^ Health assets have been described as the repertoire of potentials—internal and external strengths—that mobilize positive health behaviour and promote health and well‐being.^3^ Examples of internal strengths are optimism, sense of meaning in life, acceptance, positive emotions (eg gratitude, hope), self‐esteem and self‐efficacy.^4^, ^5^, ^6^, ^7^, ^8^, ^9^, ^10^, ^11^ Examples of external strengths include a supportive family, neighbourhood and institutions, and stable socioeconomic status.^3^, ^5^, ^6^ Using strengths in the face of chronic illness nurtures resilience, a successful adaption to adversity.^7^, ^9^ Strengths may be part of general resistance resources, a core element of salutogenesis, focusing on the individual's power to heal.^3^, ^5^, ^6^

Traditionally, patients’ strengths have not been the centre of attention in health care. The focus has been more directed towards pathology, deficits and risk factors.^14^, ^15^ Within care for people with chronic conditions, this is slowly changing with an increasing attention to strengths and resilience in social work,^14^ nursing,^16^ mental health care,^14^, ^17^, ^18^ psychology^19^, ^20^, ^21^ and medicine.^22^, ^23^, ^24^ Also, self‐management interventions are increasingly designed to empower and mobilize personal strengths, in addition to providing education.^1^, ^25^ Further, a shift in focus towards strengths is very much in line with a person‐centred care approach, a holistic view of patients and the concept of empowerment.^14^, ^26^, ^27^

Despite an increased recognition of patients’ strengths, in regular clinical practice patients’ strengths may still often be neglected by those caring for people with chronic somatic symptoms.^11^, ^28^ Awareness and activation of one's own strengths can be challenging for patients when unpleasant symptoms and emotional consequences may overwhelm and compete for attention.^13^, ^29^ Health‐care providers therefore play a crucial role in helping patients to identify and bring their attention to their strengths, which can build and broaden their capacities and empower them to take positive action.^13^, ^30^

Examples of strategies to help health‐care providers support people with chronic illness in exploring and mobilizing their strengths include training in communication techniques (such as motivational interviewing^31^ or the communication habit model^22^) and methods to assess strengths with assessment scales.^17^, ^32^ In addition, interventions aiming to prepare patients for consultations can lead to more active engagement during clinical encounters.^33^ For example, digital pre‐encounter communication interventions that help patients prepare for a conversation about their symptoms and problems were found to have positive effects on communication and consultation quality.^34^, ^35^ However, interactive digital interventions that support patients in exploring and reporting their strengths in a clinical setting are generally still lacking. Such interventions may help people become more aware and reflect on their strengths, at their own pace and convenience, in preparation for a consultation.^36^

The study presented here is part of a larger research project with the main objective of developing a digital communication intervention to help patients become aware of and engage their strengths in illness self‐management, and thus to assist patients and their care providers in building on the patients’ strengths during their consultations. While the body of literature on strengths relevant to health and well‐being is expanding, there is no systematic review or consensus on which strengths to include in such an intervention for people with chronic illness. To help patients recognize their strengths, the items in the intervention should reflect patients’ actual experiences and perceptions. The results of this study will be used to formulate examples of strengths that can be included in the digital intervention. The study reported here was thus conducted to answer the following research question: What do people with chronic illness describe as their strengths relevant to their health and well‐being?

## 2 METHODS

### 2.1 Sample and setting

The study was approved by the Regional Committees for Medical and Health Research Ethics in Norway and by the Privacy Protection Committee at the Oslo University Hospital.

A total of 39 participants from 4 outpatient rehabilitation or self‐management programmes in specialized health care were recruited. A purposive sampling procedure was used to select information‐rich participants of both genders. People with 3 different chronic conditions were included. People with these particular conditions were included for 2 reasons: (i) all required long‐term self‐management and (ii) purely practical reasons, such as access to patients whose care providers were willing to collaborate and help in recruitment. Participants included patients with chronic respiratory disease (n = 7), chronic pain conditions (n = 18) and morbid obesity (n = 14). Participants with respiratory disease were recruited from a pulmonary rehabilitation programme. Participants with chronic pain conditions were included from 2 pain clinics, and patients with obesity were recruited from a programme that was mandatory as a preparation for bariatric surgery. Inclusion criteria were as follows: (i) diagnosed with a chronic illness and being enrolled in rehabilitation or self‐management programme, (ii) >18 years old, (iii) able to speak and understand Norwegian and (iv) willing to share personal experiences about living with chronic illness. Clinicians at the recruitment sites identified eligible participants and informed them about the study. The clinicians collected names and phone numbers of those who expressed interest in participating. The authors (OBK and US) who received the lists of names contacted each person to schedule a focus group meeting or an interview. All interviews with patients were held in conference rooms at Oslo University Hospital. Participants received information about the purpose of the study, the voluntary nature of participating and maintenance of confidentiality, and gave their written informed consent. All participants were primarily invited to participate in a focus group but if they were not able to attend a focus group, they were invited to a pairwise or individual interview. In this manner, data could be obtained from all who had consented to participate. All interviews were audiotaped.

### 2.2 Procedures

The study builds on the research group's previous work on patient strengths. This includes a concept analysis,^3^ and studies of strengths from the perspective of people with or recovering from cancer^11^ and health‐care providers in cancer care.^37^ A semistructured interview guide was developed. The participants were interviewed in 4 focus groups (n = 18), in pairs (n = 6) or individually (n = 15). The individual interviews were conducted by one of the authors (OBK or US) and lasted between 30 minutes and 1 hour. In the group interviews that involved 3 or more participants, one author (US) acted as moderator and guided the interviews, which usually lasted between 1.5 and 2 hours.

The initial questions posed to participants were intentionally broad and asked: “Is there anything you have experienced or can think of as a strength or resource in your everyday life with chronic illness?” During the discussions, participants were asked probing questions to expand on what they had said, and to reflect upon strengths in themselves or their environment perceived as useful or important for their health or well‐being. The participants were also encouraged to share concrete experiences of how they used their strengths to manage chronic illness. The paired and group interviews allowed interactions among participants, who could mutually confirm, reinforce and contradict statements.

### 2.3 Analysis

Data were analysed using qualitative content analysis.^38^ In the first step of the analysis, 2 authors (OBK, US) listened through the recorded interviews to get an overview of the strengths and experiences reported. The 2 authors independently extracted and transcribed meaning units from the recorded interviews. The following question guided the extraction of meaning units: “what does the participant describe as his/her strengths?” All manifest content, that is descriptions of factors and processes reported by the participants as helpful in promoting health or well‐being, was extracted as relevant in this context. The meaning units were compared and discussed between the 2 authors and a third co‐author, a patient representative (TK). The patient representative was employed part‐time as a co‐researcher and did not know the study participants. As a second step, overlapping or similar meaning units were grouped together. This was done iteratively, by 4 of the authors (OBK, TK, US, CR). The aim was to reduce the number of strength descriptions without losing relevant meaning. To limit the risk of missing relevant units in the condensation process, one of the authors (TK) traced the strength descriptions back to the original meaning units. The results were discussed within the author group, and consensus was reached on strength descriptions representative of the extracted meaning units. Finally, the strength descriptions were categorized into 3 broad categories described below. Data management was mainly performed using Microsoft Word.

## 3 RESULTS

### 3.1 Participants

Demographic characteristics of the 39 participants (11 men) are provided in Table 1. Participants with chronic pain reported various conditions, for example fibromyalgia, arthritis, neuropathic pain and complex pain syndrome. Information about symptom severity or duration of illness was not collected.

**Table 1:** Participants’ demographic characteristics and conditions

Characteristics		% (n)
Age		
Mean age (y)	50.3	
Range (y)	31‐71	
Have children	Yes	72 (28)
Marital status	Married	56 (22)
	Unmarried/divorced/widowed	41 (16)
	Missing data	3 (1)
Highest level of education	Primary	3 (1)
	Secondary	46 (18)
	College/University	51 (20)
Employment status	Full‐time/Part‐time work	38 (15)
	Sick leave	21 (8)
	Retired/Other	38 (15)
	Missing data	3 (1)
Condition	Chronic pain	46 (18)
	Morbid obesity	36 (14)
	Chronic respiratory disease	18 (7)

### 3.2 Reported personal strengths

Reported personal strengths were categorized into 3 broad domains: (i) Internal strengths, (ii) External strengths and (iii) Self‐management strategies. In the following, examples of strengths reported within each category are described; along with illustrative examples from participants.

#### 3.2.1 Category 1: internal strengths

Descriptions of personal qualities and positive emotions were categorized as internal strengths.

##### Being persistent

A repeatedly reported strength was related to persistence. Participants described having a drive, not giving up, being stubborn and giving all they have as important positive characteristics.

When I'm at rock bottom there's only one way to go, and that's up. Then I tell myself: now you have to shape up. Now you need to move on, and you have to do it yourself. (Participant with a chronic respiratory disease)

Participants also described endurance, having a strong commitment to work despite symptoms and being self‐disciplined. Examples included continuing to do exercises learned in a rehabilitation programme years ago and being able to continue to live at home despite severe disability.

##### Having a positive outlook

Having a positive outlook and being optimistic were other important strengths reported. For instance, many participants described seeing things as challenges and not as problems, trying to find solutions or actively trying to think positive.

If I sat around thinking about everything I can't do, it would be the end of me, so I'm an eternal optimist. Instead, I try to find the positive side even when everything looks dark and hopeless. (Participant with obesity)

I get up as if I was going to work, I start my day as if I was perfectly healthy. And I try to look ahead to the day as if I was just fine, and try to function as well as I can. I feel this as a strength; that I can live with it. I made this choice when I was at my very worst. (Participant with chronic pain)

Some reported that feeling in a good mood was a strength. Participants also reported that they paid attention to things that were working well in their life despite the illness, and that being appreciative of and grateful for the good things in life, and looking forward to pleasurable events was a strength. A few participants talked about being appreciative of the benefits of being ill, for example learning to know oneself better and growing as a person.

##### Being kind and caring

Being kind, caring or helpful to others was reported as a strength by many participants. As told by one of the participants: “I'm a very caring person towards those closest to me”. Participants reported having empathy for others, being diplomatic, as well as being patient and tolerant towards others. Some participants found voluntary work to be an important strength. Kindness was reported as a source of positive emotions and a sense of meaning. Helping others was also described as a useful strategy to distract oneself from one's own difficulties.

##### Experiencing positive emotions

Participants reported the sources and experiences of various positive emotions as strengths. Joy and gratitude were commonly mentioned, but also interest, contentment and pride were described. Participants talked about positive emotions as strengths in their own rights (eg “I have lists of things I'm grateful for; there's a lot that's good too”), and also about the sources of positive emotions, for example, having supportive relationships and helping others. In addition, having a hobby or leisure activities was reported as a prominent source of positive emotions.

I had a goal that I would ski. I was at the physiotherapist and they managed to set up a program, and just that in itself was a big YES, and I got proper equipment from the store, and tips about where to find a good skiing instructor who has taught me to ski in a way I can manage. It doesn't look right, and I can't do it for very long, but I can ski for a while and I'm really pleased. I never think, like, today I'm in too much pain, I'm so exhausted, I won't make it to the slopes. No, I make it to the slopes and go home if I must. But then at least I've tried, so I'm happy and content. (Participant with obesity)

Spending time in nature was mentioned by several participants as a strength. As one participant said: “Nature helps me a lot. Seeing, being present, smelling, going on a hike. I'm lucky that way”.

Also, engagement in work or education was a reported strength. Further, some said that having a sense of humour was helpful in difficult situations. One participant talked about the importance of religious faith.

##### Being kind towards oneself

Some participants reported strengths that reflected kindness towards oneself. Descriptions from participants included viewing oneself with kindness, being less self‐critical and not comparing oneself with others.

I've started to be more loving toward myself, don't ask too much of myself, I'm good enough as I am. (Participant with chronic pain)

Several participants talked about giving themselves enough time to do things in their own pace, listening to their bodies, living in the present moment and trying to rest and avoid stress. Many participants reported giving themselves permission to prioritize themselves and to set aside time for self‐care and rest as examples.

I don't just say yes all the time anymore. I stop and think a bit more when people make requests or want me to join in or to do something. […] What's in it for me? I used to say yes to everything. (Participant with obesity)

##### Reconciling oneself with the situation

Some participants reported the process of reconciling oneself with or accepting symptoms and limitations of their condition as a strength.

I've found my place in life, reconciled myself with my situation. (Participant with chronic pain)

The pain will always be with you, as a monster or as a friend depending on how you choose to see it; it's a part of you. The pain has become part of me whether I like it or not. I don't know how I would have managed to handle the pain if I hadn't accepted that. (Participant with chronic pain)

Several participants reported that adjusting their ambitions and goals according to their condition and current situation was important.

##### Having courage

Some participants provided descriptions that suggested some level of courage, for example willingness to try out new things, showing vulnerability and asking for help. As told by a participant with chronic pain: “I dare to accept help instead of being good and trying to manage on my own”. Setting boundaries towards others was described, for example being selective about whom to share illness‐related information with. Nevertheless, several participants mentioned openness as a strength and that being able to share their experience was helpful. Assertiveness and the courage to speak out were reported as strengths, for example in order to get needed help from health‐care providers or as a response to condition‐related stigma.

I could choose to just lie down in bed, keep to myself, but my senses tell me that this is not good […], I have the courage to be in front of other people and be seen and heard. (Participant with obesity)

##### Having knowledge and insight

Belief in one's own ability to manage symptoms and obtaining a sense of control was reported as another strength. This was supported by having the necessary knowledge to manage the condition, for example knowledge about the medical regimens and suitable exercise types. Several participants mentioned the importance of awareness of the body‐mind relation, for example the importance of rest and how stress influences their symptoms. The participants provided different examples of how knowledge and insight reduced distress and promoted a sense of control.

Knowledge and insight into my own condition made me more secure in handling it in a different way. Now I can take precautions and adjust a bit how I take my medicines. Then I feel more secure, calmer, and more in control of what happens in my chest. Things are more predictable, my anxiety about freezing up and not breathing, it disappears when I'm in control. (Participant with a chronic pulmonary condition)

I think a lot of the pain I've been suffering from has been psychosomatic or whatever it's called, so when I got the psychological stuff under control and did away with a lot, or got help and a few techniques to deal with some of my mental challenges, since then I haven't had as much pain. I used to have pain in my whole body, and now I don't. It only hurts in my shoulder, and I think that's because of working at the computer. (Participant with chronic pain)

#### 3.2.2 Category 2: external strengths

Many reported supportive relationships and support from health‐care providers as a strength. Relationships with family and friends were seen as highly important for emotional and practical support, including support to engage in positive health behaviour. They valued friends and family members that were aware of the changes caused by the illness, understood their limitations, acknowledged their health problems and respected their situation. The participants also valued being together with peers, and some found it easier to share their thoughts and experiences related to their illness with peers rather than family and friends. They found it rewarding to talk to people who had been through similar experiences. Fellowship with peers reduced the feeling of being alone associated with having a chronic illness.

It helps to talk to others about everything you can't manage; it also makes it easier to be satisfied with yourself because you hear that other people are just the same. You're not the only one. (Participant with chronic pain)

Many described having access to trustworthy health‐care providers as important. Also, surroundings that supported a healthy lifestyle were reported as valuable, for example having access to a gym at the workplace or joining others in a training programme. Living in a friendly and safe neighbourhood was reported as a strength as was financial security.

#### 3.2.3 Category 3: self‐management strategies

When participants were asked about their strengths, they also reported a variety of strategies that helped them promote health and well‐being. Examples of strategies that were commonly mentioned were as follows: be active, exercise, reduce stress and get enough rest. Other strategies were as follows: to take things slowly, plan and prioritize, set and adjust goals, and work towards these goals.

I have to plan my every day, to make sure it doesn't get too much, otherwise I just have to cancel things. (Participant with chronic pain)

Participants mentioned a number of strategies to manage symptoms, including reflective writing, distraction, breathing exercises and visualization.

An ability to visualize, imagine myself in various situations, and it turns into like a daydream and gives me some comfort. For instance, I have an ability to feel slim, and I can use that a bit to move around as though I was slim, so then I do a double‐take every time I see my reflection in a window or a mirror. But it gives me some sort of satisfaction, just like I really believe it a bit, inside my own head. (Participant with obesity)

When I'm in a lot of pain, I try to find something inside my head that will engage me, something I'd like to do, instead of sitting there thinking about how much it hurts. Try to shift focus. (Participant with chronic pain)

Participants also talked about strategies for handling stress, such as seeking knowledge and help when needed, various methods for managing difficult thoughts, using humour, as well as seeking out people with similar experiences and concerns to share with and learn from.

## 4 DISCUSSION AND CONCLUSION

### 4.1 Summary of results

The study provides examples of how strengths are perceived and reported in the context of chronic illness. Participants described a broad repertoire of personal strengths that we categorized as internal and external strengths and self‐management strategies. Internal strengths included persistence, positive outlook, kindness towards oneself and others, acceptance, courage, positive emotions and also knowledge and insight. External strengths included supportive relationships with family, friends, peers and health‐care providers. Self‐management strategies included keeping active, planning and prioritizing, reducing stress, goal setting and seeking knowledge and help.

### 4.2 Comparison with prior research

#### 4.2.1 Personal strengths

The present study indicates that when asked about their strengths, and with appropriate prompts, people with chronic illness report processes similar to that have been described in the literature to promote successful self‐management and resilience. These include knowledge and insight, positive emotions, optimism, sense of purpose, active coping and social support.^2^, ^7^, ^9^ In a study on personal strengths in people with or recovering from cancer, good mood, mindfulness, willpower, positive relations, hopes and beliefs, protection and taking action and control were reported as strengths.^11^ Our results are very much in line with these findings. However, some differences were found as people with chronic illness in the present study also described acceptance, kindness and care, as well as a range of positive emotions as strengths.

When asked about their strengths, participants did not only talk about internal or external qualities but also about their self‐management strategies or behaviour (such as health‐promoting activities and behaviour promoting positive emotions). Health behaviour can be viewed as a consequence of mobilization of personal strengths.^3^ However, as the objective of the study was to describe what patients’ themselves report as their strengths and their description included self‐management strategies and behaviour, these were included in the results as a separate category. Supportive relationships were categorized as external strengths together with environmental resources. In a review of assessment instruments of strengths for use in mental health care, items were categorized into 3 main domains: individual, environmental and interpersonal.^17^ Arguably, social support involves interpersonal strengths and could have been categorized as a separate domain.

#### 4.2.2 Salutogenesis

Salutogenesis is a widely used theoretical construct underlying personal strengths and health assets.^3^ The salutogenic approach contributed to a shift from a pathogenic focus on risk factors for diseases to a focus on salutary factors, that is strengths and determinants for health.^12^ In salutogenesis, the major salutary factors, referred to as the generalized resistance resources (GRRs), have been specified as biological, material and psychosocial factors, for example money, knowledge, experience, social support, culture and intelligence.^39^ The degree of overlap between GRRs and personal strengths depends partly on how health is defined. The current study applied a definition of strengths that includes both health and well‐being as outcomes.^3^ In the research field of salutogenesis, it is debated if the concept of health should include well‐being or not.^40^ Regardless, the results of the present study build upon the knowledge about GRRs in people with chronic illness. In particular, it adds to knowledge about subjective experience of salutary psychosocial factors, as well as providing examples of the application of personal strengths in people with chronic illness.

#### 4.2.3 Positive psychology

Recently, an integrative approach that combines salutogenesis and positive psychology has been proposed.^41^ The present findings include several strengths similar to items from the taxonomy of character strengths in positive psychology, that is perseverance, kindness, bravery, humour and gratitude.^42^ However, most studies on character strengths have included participants from the general population.^43^ The present study therefore adds to knowledge about the strengths and their applicability reported by people with chronic illness. For example, participants in the present study reported kindness towards oneself as an important strength. Self‐kindness is, however, not included in the taxonomy of character strengths (qualities of compassion are included but only in relationships with others, ie love and kindness^42^). Self‐kindness refers to the ability to treat oneself with kindness rather than judgement during challenging situations, and it is an essential component in self‐compassion.^44^ In people with chronic conditions, self‐compassion has been linked to lower stress levels^45^, ^46^, ^47^ suggesting its role in self‐management. Courage, one of the character strengths,^42^ was also reported by our participants as a strength. Cultivating courage is considered an important part of activating psychological resources to promote constructive self‐management of chronic illness.^2^

#### 4.2.4 Self‐management and external strengths

Several of the internal strengths reported by participants in this study, like persistence, positive outlook, acceptance and knowledge, have previously been described as important self‐management processes for people with chronic illness.^2^, ^48^ For example, persistence may be related to cultivating discipline and motivation, essential for successful self‐management.^2^ Also, positive emotions and its various sources were described as important strengths. This too is in accordance with prior self‐management and resilience research, indicating the importance of positive emotions for promoting health.^2^, ^7^, ^9^, ^49^

The strengths categorized as self‐management strategies confirm a large body of literature on self‐management, for example health promotion activities, seeking support, goal setting, planning and pacing.^2^ External resources included the support from family and friends as well as health‐care resources. The availability of emotional and practical support was an important strength. This is in line with the well‐documented importance and effect of social resources reported in prior research.^2^, ^3^

### 4.3 Practical implications

Becoming aware of and utilizing personal strengths have been established as an important process for people to live well with chronic illness.^2^ This study extends the understanding of the subjective appraisal and application of personal strengths in people with chronic illness. Supporting mobilization of patients’ personal strengths in order to promote optimal self‐management and well‐being is important to integrate into health care for people with chronic conditions.^16^, ^22^, ^50^ The study results provide examples of strengths that can be useful for health‐care providers to recognize and nurture in their patients. By asking about their personal strengths, health‐care providers can help patients acknowledge and further build on these strengths to promote health and well‐being.

### 4.4 The study's strengths and limitations

The credibility of the findings is supported by several factors. The high number of participants allowed for some saturation in findings; and the team involved in the coding and categorization included a patient representative. Descriptions of strengths are highlighted with quotations from the informants. For practical reasons, interviews were carried out individually or in a group; however, combining individual interviews and focus groups may have contributed to enhanced data richness.^51^

The findings must also be interpreted in the context of the study's limitations. First, the interpretation of relevance and meaning is unavoidably influenced by the researchers’ backgrounds.^38^ Second, the study was an early step in a person‐centred design of an intervention. Due to limited resources, some steps that could have increased the quality of the analysis were left out, for example verbatim transcription of complete audio data and checking of inter‐rater reliability between coders. Participants were recruited from self‐management programmes, and people without this experience might depict their strengths in a different way or be less aware of them. Some participants did need more prompting than others and some participants talked about their perceived lack of strengths. The participants’ level of education should also be considered, as most of the participants had education beyond primary level. Last, but not least, people with different conditions were included, but differences between strengths described by people with different diagnoses were not explored.

## 5 CONCLUSION

The study amplifies and expands upon previous findings on personal strengths in people with health challenges and adds emphasis on persistence, positive outlook, kindness towards self and others, acceptance, courage and positive emotions. By becoming more knowledgeable about the richness of strengths patients use, health‐care providers can play an important role as catalysts for what patients pay attention to and help them become more aware of and cultivate their strengths to promote health and well‐being. Supporting patients in mobilizing their strengths is essential in providing empowering person‐centred care for people with chronic illness.

## CONFLICT OF INTEREST

No conflict of interest.
